# Enzyme Inhibitory Activities of Extracts and Carpachromene from the Stem of *Ficus benghalensis*

**DOI:** 10.1155/2022/7053655

**Published:** 2022-12-20

**Authors:** Abdur Rauf, Muhammad Ibrahim, Naveed Muhammad, Saima Naz, Abdul Wadood, Bilal Khan, Toseef Ali, Muhammad Saleem, Abdulrahman Alsahammari, Metab Alharbi, Hafiz Ansar Rasul Suleria

**Affiliations:** ^1^Department of Chemistry, University of Swabi, Swabi, Anbar, 23430 Khyber Pakhtunkhwa (KP), Pakistan; ^2^Department of Pharmacy, Abdul Wali Khan University, Mardan, Khyber Pakhtunkhwa (KP), Pakistan; ^3^Department of Biotechnology, Bacha Khan University, Charsadda, Khyber Pakhtunkhwa (KP), Pakistan; ^4^Department of Biochemistry, Abdul Wali Khan University, Mardan, Khyber Pakhtunkhwa (KP), Pakistan; ^5^Department of Chemistry, Ghazi University, Dera Ghazi Khan, 32200 Punjab, Pakistan; ^6^Department of Pharmacology and Toxicology, College of Pharmacy, King Saud University, Post Box 2455, Riyadh 11451, Saudi Arabia; ^7^School of Agriculture and Food, Faculty of Veterinary and Agricultural Sciences, The University of Melbourne, Parkville, Victoria 3010, Australia

## Abstract

*Ficus benghalensis* is one of the potential medicinal plants which is used locally for the treatment of various ailments such as diabetes, antiasthmatic, and wound healing. To provide a scientific background to these folklores, the current study was designed to evaluate the extract and isolated compound against various enzymes such as ureases, tyrosinase, and phosphodiesterase. The methanolic extract and carpachromene demonstrated a significant urease inhibition effect with maximum percent inhibition of 72.09 and 92.87%, respectively. Regarding the tyrosinase inhibition, the percent antagonist effect of carpachromene and the methanolic extract was 84.80 and 70.98%, respectively. The phosphodiesterase was also significantly antagonized by crude extract and carpachromene with a maximum percent inhibition of 82.98% and 89.54%, respectively. The docking study demonstrated that the carpachromene fits well into the active site of all three enzymes with significant interactions. Carpachromene might possess the potential to inhibit all three enzymes and can effectively treat different diseases associated with the hyperactivity of these enzymes. In conclusion, the crude extract and carpachromene exhibit significant urease, tyrosinase, and phosphodiesterase inhibitory activity which might be used against various diseases. In conclusion, the crude extract and carpachromene exhibit significant urease, tyrosinase, and phosphodiesterase inhibitory activity which might be used against diabetes and bronchoconstriction. Further, the current study provides scientific backup to the folklore (antidiabetic and antiasthmatic) of *Ficus benghalensis*.

## 1. Introduction

Biodiversity is approved with the remedial plant's permanent resource, which provides mankind with a loaded source of drugs [1]. More than 80% of people depend on traditional drugs as a concern for their primary health care. The remedial plant is consumed for thousands of years and is well known for its success in the different treatments [2]. The usages of medicinal plants and their extracts were documented in a biochemical report. The prepared herbal medicine is mostly herbal medicinal [3]. The remedial plants provided the raw material for drug formulation such as Unani, Ayurveda, and Siddha in modern medicines [4]. The remedial plant does not give only medicine for the treatment of various diseases but is also used in preparing herbal products and cosmetics [5]. Due to deforestation and the overutilization of the forests for various purposes, the remedial plant sources are depleting, and calls for the conservation of the remedial plant are germplasm resources. Resources of conservation can be done by creating a great scale of awareness of the importance of therapeutic plants [6].

*Ficus benghalensis* is an evergreen higher plant. *Ficus benghalensis* is a member of the family Moraceae [7]. This plant is generally known as the “Indian banyan tree.” The tree banyan also contains numerous religious and mythological atmospheres [8]. The leaves are simple and option, the size ranges from 1 to 20 cm in length and 5 to 12.5 cm wide, and the veins are 7 to 8 pairs. The flower is minute and unisexual of three kinds, females, males, and faulty females [9]. The male flower is close to the mouth of the vessel, perianth [10]. A remedial property of the different parts of the plant has been famous to the local physician. The milky fluid of the *Ficus benghalensis* is useful on the outside for pains, rheumatism, bruise and backache, and fractured of swollen soles of the feet [10]. In India, the *Ficus benghalensis* roots are utilized for the treatment of dysentery, biliousness, gonorrhea, and swelling of the liver. The aerial roots of tips are used for curing dysentery and vomiting. Small branches of the infusion are consumed for the hemoptysis while the bark is imaginary to be a powerful tonic and used in the treatment of diabetes [11]. Various parts of *Ficus benghalensis* are used for the treatment of skin disease, wound healing, leucorrhea, eye disease, diabetes, and diarrhea [12]. In the folkloric system, the aerial parts of *Ficus benghalensis* are used for curing persisting vomiting and as antiasthmatic [13–15]. *Ficus benghalensis* leaves and stem are also reported for the treatment of various diseases [16–18]. Phytochemically, this plant has been explored very little; its leaves contain rutin, *β*-amyrin, and leucopelargonon, along with *β*-sitosterol, psoralen, quercetin-3-galactoside 20, leucodelphinidin derivative, glucoside, and leucodelphinidin derived [19]. Based on the medicinal importance of *Ficus benghalensis*, this project is aimed at exploring it phytochemically and pharmacologically, keeping in view the antidiabetic and bronchodilator effects of *Ficus benghalensis.* This study deals with the isolation and *in vitro* enzyme inhibitory screening of extracts and isolated carpachromene from the stem of *F. benghalensis*.

## 2. Material and Methods

### 2.1. Plant Collection

The stem of *Ficus benghalensis* was collected in June 2020, from Anbar Swabi, KP, Pakistan. The plant specimen was identified by Dr. Muhammad Ilyas, Department of Botany, University of Swabi, Pakistan; a specimen No. UOS-BOT/103 was deposited in the herbarium of the Botany Department, University of Swabi, KP, Pakistan.

### 2.2. Extraction and Isolation

The collected stem of *Ficus benghalensis* was dried under shade for 24 days. The dried plant material was converted to powder. The powder stem (2.00 kg) of *Ficus benghalensis* was extracted with methanol, to obtain the methanolic extract (24.50 g). The methanol extract was fractionation into *n*-hexane and ethyl acetate fractions with the help of the Soxhlet apparatus. The ethyl acetate extract (20 gm) was subjected to column chromatography using silica gel. The column was eluted with chloroform and methanol (80 : 20), which yield 20 subfractions (BK1-BK20). Based on the TLC profile, BK4 was subjected to repeated column chromatography using silica gel. The column was eluted with chloroform and methanol (92 : 08) which afforded compound 1 (1.69 gm and 99.95% pure).

### 2.3. Urease Assay

The urease assay was performed using the spectrometry method (96-well plate) [20]. For this assay, 25 *μ*l urease enzyme (1 *μ*l/well) and extract/carpachromene (0.2 *μ*g/ml) were processed at the recommended temperature for 15 min. After the above treatment, 55 *μ*l urea was added and the mixture was incubated at 30°C for 15 min. This mixture was then supplemented with 45 *μ*l of phenol (0.005% *w*/*v* Na-nitroprusside and phenol 1% *w*/*v*), and 70 *μ*l of alkali reagents (0.1% Na-hypochlorite and 0.5% *w*/*v* NaOH) was added. The supplemented plates were inoculated for 50 min at the same temperature. Urease inhibitory potential was determined with constant urea breakdown and NH_3_ production. The urease inhibition potency of extracts and the isolated carpachromene were compared with thiourea (standard). The significant variation in absorbance (optical density (OD)) was determined at 630 nm using an ELISA plate reader.

### 2.4. Tyrosinase Assay

The samples to be tested were performed as that extract or carpachromene (0.2 *μ*l/ml) was added to 96 well microplates and was mixed out with phosphate buffer solution (60 *μ*l) and 10 *μ*l (*μ*/well) mushroom tyrosine in 30 *μ*l phosphate buffer. After inoculation of this reaction mixture at a temperature of 37° for 30 min, 20 *μ*l of L-DOP in phosphate buffer was mixed and absorbance was determined spectrophotometrically at 480 nm. The tyrosinase inhibition potential of extracts and carpachromene was comparable with the positive control drug (kojic acid). The percent activity was calculated through a published procedure [21]. The percent inhibition of the enzyme was calculated using an MS Excel®™ 2000 (Microsoft Corp., USA) program, developed for this purpose: percent inhibition = [ABS_Blank_ − (ABS_Sample_/ABS_Blank_)] × 100.

### 2.5. Phosphodiesterase Assay

The samples to be tested were subjected to a phosphodiesterase (PDE) assay [22]. The assay was performed by combining 97 *μ*l of Tris hydrochloric acid buffer with a pH of 8.8 and 20 *μ*l of magnesium acetate (30 mM) to 96-well plates. The above mixture was supplemented with extract or carpachromene (0.2 *μ*g/ml) and 15 *μ*l (1 *μ*l/well) of PDE-1 as the final concentration of 0.000742 U/well, and the mixture was incubated in ELISA at a temperature of 37°C for a period of 30 min. In this assay, magnesium acetate was mixed as PDE-1 cofactor. After the above treatment, 60 *μ*l of the substrate in the form of 0.33 mM bis (p-nitrophenyl) was added. The inhibitory potential of samples was monitored at 410 nm on a microplate reader for 30 min duration. The phosphodiesterase (PDE) inhibitory activity of the extracts and carpachromene was compared with EDTA (standard). The inhibitory activity of substances is determined in the form of % inhibition. It is calculated by using the following formula:

(1) $$\begin{array}{c} \%\text{Inhibition}=100-\left(\frac{\text{OD test well}}{\text{OD control}}\right)\times 100. \end{array}$$

The EZ-fit enzyme kinetics software from Perrella Scientific (Amherst, NH, USA) was used to calculate the IC_50_ values.

### 2.6. Molecular Docking

Molecular docking was performed to study the binding mode of the carpachromene in the active site of urease, tyrosinase, and phosphodiesterase. The crystal structures of tyrosinase urease (PDB ID: 4H9M), an oxy form of tyrosinase (PDB ID: 1WX2), and phosphodiesterase 1B (PDB ID: 5W6E) were taken from the Protein Data Bank. The MOE (2016) software was used to carry out molecular docking studies. The structure of the target was prepared in MOE as well [23]. The energy was reduced up to 0.05 gradient followed by 3D protonation. Before docking, all the water molecules were excluded from the structure [24]. Using the Triangular Matching docking method, the isolated compound was docked into the active site of all three receptors. Ten conformations were generated for the isolated compound. The MOE's dG docking score tool was used to calculate the binding free energy between the ligand and the receptor (lower values suggest stronger binding affinity) [25]. The PyMOL software was used for the visualization of protein-ligand interactions [26].

## 3. Results

### 3.1. Structure Elucidation of Compound 1

**Figure 1:**
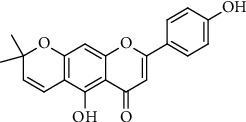
Chemical structure of carpachromene isolated from *Ficus benghalensis*.

The structure of carpachromene was elucidated by advanced spectroscopic technique. Carpachromene was extracted in yellow amorphous powder from the ethyl acetate soluble extract of *Ficus benghalensis*. ESI-MS exhibited m/z 337 (M+H) demonstrating the molecular formula C_20_H_16_O_5_. ^1^HNMR ((DMSO; 600 Mz) *δ*_H;_ 1.48 (6H, s, H-4^//^, H-5^//^), 5.77 (1H, d, J=9.8 Hz, H-2^//^), 6.53 (1H, s, H-8), 6.55 (1H, d, J=10.5 Hz, H-1^//^), 6.80 (1H, s, H-3), 6.92 (2H, d, J=8.7 Hz, H-3/, H-5/), 7.90 (2H, d, 8.8 Hz, H-2^/^, H-6^/^respectively. ^13^ C-NMR (DMSO; 300 Mz) 183.1 (C-4), 164.3 (C-7), 162.1 (C-5), 158.8 (C-2) 156.8 (C-4^/^), 155.7 (C-9), 128.9 (C-2^//^), 128.7 (C-2^/^), 121.4 (C-1^/^) 115.8 (C-3^/^, C-5^/^), 114.9 (C-1^//^), 105.1 (C-6), 104. 8 (C-10), 102.8 (C-3), 95.2 (C-8), 77.9 (C-3^//^), 27.8 (C-4^//^), 27.8 (C-5^///^), respectively. The structure of compound 1 (Figure 1) was identified by comparing the spectroscopic data with previously reported data [20, 27].

### 3.2. Urease Inhibition

The extracts and carpachromene were tested for urease inhibitory potential. The results are displayed in Table 1. The maximum urease inhibition was observed against carpachromene (92.87%) followed by methanolic extract (72.09%) with IC_50_ values of 27.09 and 90.32 *μ*M, respectively.

### 3.3. Tyrosinase Inhibition

The extracts and carpachromene demonstrated a variable degree of tyrosinase inhibition as shown in Table 1. The maximum percent inhibition was noted against carpachromene (84.80) and methanolic extract (70.98%). The tyrosinase inhibition potential of carpachromene was comparable with the positive control drug (kojic acid).

### 3.4. Phosphodiesterase Inhibition

The samples to be tested were also effective against phosphodiesterase as shown in Table 1. The minimum inhibitory effect was noted against *n*-hexane (40.76%) while ethyl acetate (79.12%). Methanol and carpachromene demonstrated 79.12, 82.98, and 89.54% effects, respectively.

### 3.5. Docking Study Analysis

**Figure 2:**
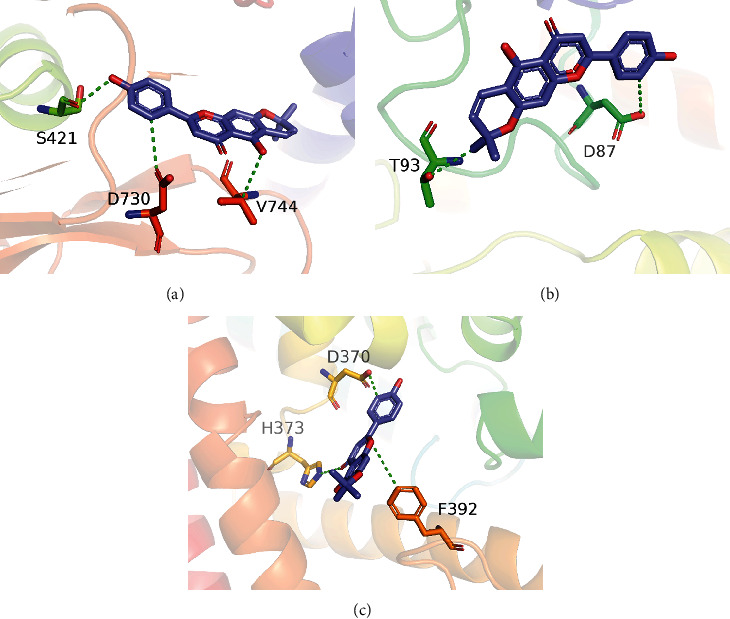
3D representations of carpachromene isolated from *Ficus benghalensis* against urease, tyrosinase, and phosphodiesterase: (a) urease, (b) tyrosinase, and (c) phosphodiesterase. Amino acids are represented by one letter code: D: aspartic acid; F: phenylalanine; H: histidine; S: serine; T: threonine; V: valine.

The isolated compound was docked to urease, tyrosinase, and phosphodiesterase, to estimate the best binding pose. The docking score (kcal/mol) and bonding interaction pattern (hydrogen) were used to investigate the docked complexes. Molecular docking analysis revealed that the docking score of phosphodiesterase and isolated compound-complex was −6.732 followed by urease-compound-complex −6.531. The docking score of tyrosinase and compound-complex was −6.215.  Table 2 shows the docking score and interactions of all three receptors with the isolated compound. When the compound was docked into the active site of the phosphodiesterase enzyme, it was observed that the compound formed one H-donor interaction with ASP 370, one H-acceptor interaction with HIS 373, and one pi-H interaction with PHE 392 key residues. The docking study of urease and compound revealed that the compound made two H-bond donor interactions with ASP 730, SER 421, and one H-bond acceptor interaction with VAL 744 active site residues. In the case of tyrosinase and isolated compound, it was observed that the compound made two H-donor interactions with THR 93 and ASP 87. Figures 2(a)–2(c) show the 3D interactions of the carpachromene in complex with urease, tyrosinase, and phosphodiesterase.

## 4. Discussion

The use of plant-based remedies is increasing among the world population due to easy availability and affordability [28, 29]. Therefore, researchers are in a continuous struggle to find significant natural-based remedies used for the treatment, diagnosis, mitigation, or prophylaxis of various disorders [30]. The confirmation of therapeutic potentials in these locally available natural products will provide a cheaper therapeutic option, especially within the current high inflation rates around the globe. The pharmacodynamics of most of the drugs is related to enzyme inhibition available in various biological compartments. Therefore, we tested our crude extract, fractions, and isolated compound of *Ficus benghalensis* against different biological enzymes. The results of urease enzyme indicated that methanolic extract and carpachromene showed promising inhibition. The urease is one of the notorious enzymes for the induction of *H. pylori*, urinary tract infection, and even hepatic encephalitis [31]. The significant antagonistic effect of crude extract and isolated compounds reflects therapeutic potential in this infection. Interestingly, the *Ficus benghalensis* is locally used against live inflammation and urinary tract infection [32, 33]. So the urease inhibitory effect supports these folklores. *H. pylori* is treated with multiple antibiotics for longer times which are associated with side effects and poor patient compliance. To avoid polypharmacy and improved compliance, the search for new, effective, and safe compounds is very essential. Therefore, the further mechanistic study is of utmost essential up to clinical trials. The significant antagonistic effect of methanolic extract and carpachromene against tyrosinase indicates the cosmeceutical effect of these samples. The tyrosinase inhibitors such as kojic acid, azelaic acid, and hyderquin are topical whiting and antipigment dosage form [34]. In the field of cosmetics, a lot of new alternatives are the need for the day because of the side effects of available therapeutic agents such as skin irritation [34, 35]. Therefore, the application of this natural product might be safe as compared to available synthetic compounds. This study suggests finding the cosmeceutical role of these samples in various skin models. The phosphodiesterase inhibitory effect of our tested extract and carpachromene reflects the use of this plant in various conditions such as antiasthmatic [13], vasodilator, and erectile dysfunction. Furthermore, the inhibitory activity of the isolated compound of *Ficus benghalensis* against urease, tyrosinase, and phosphodiesterase was evaluated by molecular docking, the docking results are in agreement with the experimental activity, the compound docked well in the active site of urease forming three hydrogen bonds, and the best interaction of the compound with the target protein reflects the best inhibitory activity of compound toward the target protein, compared to urease and phosphodiesterase compound forming less hydrogen bond interaction with tyrosinase. Overall, the isolated compound formed significant interactions with all the target enzymes and has the potential to combat the disease associated with this enzyme.

## 5. Conclusion

In conclusion, the crude extract and isolated compound showed significant urease, tyrosinase, and phosphodiesterase inhibitory potential. Furthermore, the molecular docking studies also confirmed the binding mode of the isolated compound in the active site of urease, tyrosinase, and phosphodiesterase. The docking study shows that the isolated compound fits well into the active site of all three enzymes and makes strong bonds with all three enzymes. Our compound might possess the potential to inhibit all three enzymes and effectively treat different diseases associated with these enzymes. The identified inhibitors can act as lead compounds for the discovery of new treatments for the diseases associated with the disorder of said enzyme.

## Figures and Tables

**Table 1:** Enzyme inhibitory screening of extracts and carpachromene isolated from *Ficus benghalensis*.

Enzyme	Tested extracts/compound	% inhibition	IC_50_ ± SEM (*μ*M)
Urease	Hexane	45.76 ± 1.98	—
	Ethyl acetate	67.09 ± 1.54	119.09 ± 1.09
	Methanol	72.09 ± 1.98	90.32 ± 1.65
	Carpachromene	92.87 ± 1.54	27.09 ± 1.65
	Thiourea	97.99 ± 1.65	21.09 ± 1.07

Tyrosinase	Hexane	39.01 ± 1.22	—
	Ethyl acetate	59.43 ± 1.00	106.09 ± 2.09
	Methanol	70.98 ± 1.03	86.09 ± 2.98
	Carpachromene	84.80 ± 1.25	40.54 ± 2.00
	Kojic acid	86.08 ± 1.09	43.98 ± 1.10

Phosphodiesterase	Hexane	40.76 ± 1.43	—
	Ethyl acetate	79.12 ± 1.43	195.32 ± 1.09
	Methanol	82.98 ± 1.87	198.43 ± 1.43
	Carpachromene	89.54 ± 2.00	209.43 ± 1.87
	EDTA	87.32 ± 1.78	264.09 ± 1.09

**Table 2:** Docking score and interactions of the carpachromene with the three receptors.

Target protein	Interacting residues	Interaction type	Distance	*E* (kcal/mol)	Docking score
Urease	ASP 730	H-donor	3.46	−0.2	−6.531
	SER 421	H-donor	2.85	−1.4	
	VAL 744	H-acceptor	3.75	−0.2	

Tyrosinase	THE 93	H-donor	3.36	−0.1	−6.215
	ASP 87	H-donor	3.67	−0.4	

Phosphodiesterase	ASP 370	H-donor	2.79	−0.5	−6.732
	HIS 373	H-acceptor	3.84	−0.4	
	PHE 392	pi-H	3.93	−0.2	

## Data Availability

The data related to this paper is given in the main text of this paper.
